# Supplementary material for: Endometrial receptivity and implantation require uterine BMP signaling through an ACVR2A-SMAD1/SMAD5 axis
Source: Nat Commun. 2021 Jun 7;12:3386. doi: 10.1038/s41467-021-23571-5 (PMC8184938; doi:10.1038/s41467-021-23571-5)
Supplement: Supplementary file 1 — Supplementary information [file 41467_2021_23571_MOESM1_ESM.pdf]

SUPPLEMENTARY INFORMATION

Endometrial receptivity and implantation require uterine BMP signaling through an ACVR2A-SMAD1/SMAD5 axis

Diana Monsivais[1,2,#], Takashi Nagashima[1,*], Renata Prunskaite-Hyyryläinen[3], Kaori Nozawa[1,2], Keisuke Shimada[4], Suni Tang[1], Clark Hamor[1], Julio Agno[1,2], Fengju Chen[5], Ramya Masand[1], Steven L. Young[6], Chad J. Creighton[5,7], Francesco J. DeMayo[8], Masahito Ikawa[4], Se-Jin Lee[9,10], and Martin M. Matzuk[1,2,#]

[1]Department of Pathology & Immunology, Baylor College of Medicine, Houston, TX 77030
[2]Center for Drug Discovery, Baylor College of Medicine, Houston, TX 77030
[3]Faculty of Biochemistry and Medicine, University of Oulu, Oulu, Finland
[4]Research Institute for Microbial Disease, Osaka University, Osaka, Japan
[5]Department of Medicine, Baylor College of Medicine, Houston, TX 77030
[6]Department of Obstetrics and Gynecology, University of North Carolina School at Chapel Hill, Chapel Hill, NC 27599
[7]Dan L. Duncan Comprehensive Cancer Center, Baylor College of Medicine, Houston, TX 77030
[8]National Institute of Environmental Health Sciences, Research Triangle Park, NC 27709
[9]Jackson Laboratory for Genomic Medicine, Farmington, CT 06032
[10]University of Connecticut School of Medicine, Department of Genetics and Genome Sciences, Farmington, CT 06030

[*]Takashi Nagashima is presently at the Hanakoganei Ladies Clinic, 1-19-3 Minamicho, Kodairashi, Tokyo, 187-0003 Japan. Tel/Fax: +81-42-497-5218/+81-42-497-5219

[#]Corresponding authors. These authors jointly supervised this work.

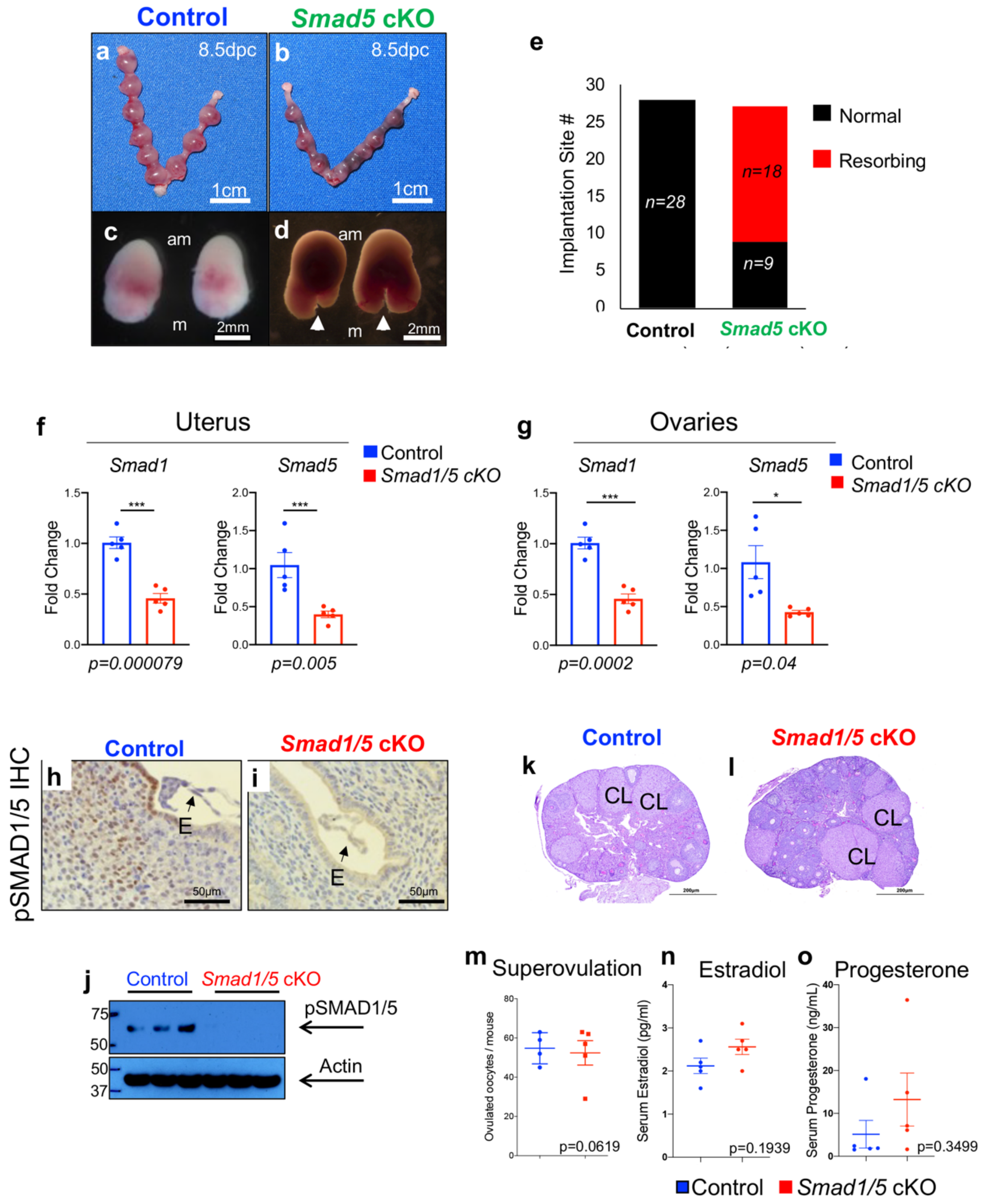

Supplementary Figure 1. Single *Smad5* cKO defects in decidualization and ovarian histology, superovulation studies and serum hormone analyses show normal ovarian function in *Smad1/5* cKO mice. a-b) 8.5dpc implantation sites from control (**a**) and *Smad5* cKO (**b**), with arrows indicating hemorrhagic and resorbing implantation sites. **c-d**) Dissected 8.5dpc implantation sites from control (**c**) and *Smad5* cKO (**d**) mice, arrows indicate the defective partially decidualized stroma. (m, mesometrial; am, anti-mesometrial) arrowheads in (**d**) indicate defective decidual regions. **e**) Quantification of normal

and resorbing implantation sites form control (n=3) and *Smad5* cKO (n=3) mice. **f-g**) qPCR analysis of *Smad1* and *Smad5* exon 2 in the uterus (**f**) or ovaries (**g**) of control and *Smad1/5* cKO mice. Histograms represent mean ± standard error of the mean (SEM). Paired, two-tailed, student's *t*-test*,* *p<0.033, **p<0.002, ***P<0.001. **h-i**) pSMAD1/5 IHC in the uterus of control (**h**) and *Smad1/5* cKO (**i**) mice at 4.5dpc. *E*, embryo. Representative image of the embryo, observed in at least 3 specimens from different mice. **j**) Western blot of uterine tissues from control and *Smad1/5* cKO mice, numbers on blot indicate protein molecular weight markers (kilodaltons, kD). IHC and qPCR analyses were performed in at least 3 samples per genotype. **k-l**) PAS stain of ovarian cross-sections from randomly cycling control (**k**) and *Smad1/5* cKO (**l**) mice show the presence of follicles and corpora lutea (CL) in both genotypes. Size-bar is 200µm. **m**) 3-week-old control and *Smad1/5* cKO mice were treated with PMSG+hCG to trigger superovulation followed by quantification of ovulated oocytes. **n-o**) Serum levels of estradiol (**n**) and progesterone (**o**) were quantified in randomly cycling 12-week-old control (n=5) and *Smad1/5* cKO (n=5) mice. Histology was performed in at least 3 samples per genotype. Histograms in (**n-o**) represent mean ± standard error of the mean (SEM), unpaired, two-tailed, student's *t*-test, *p<0.033, **p<0.002, ***P<0.001.

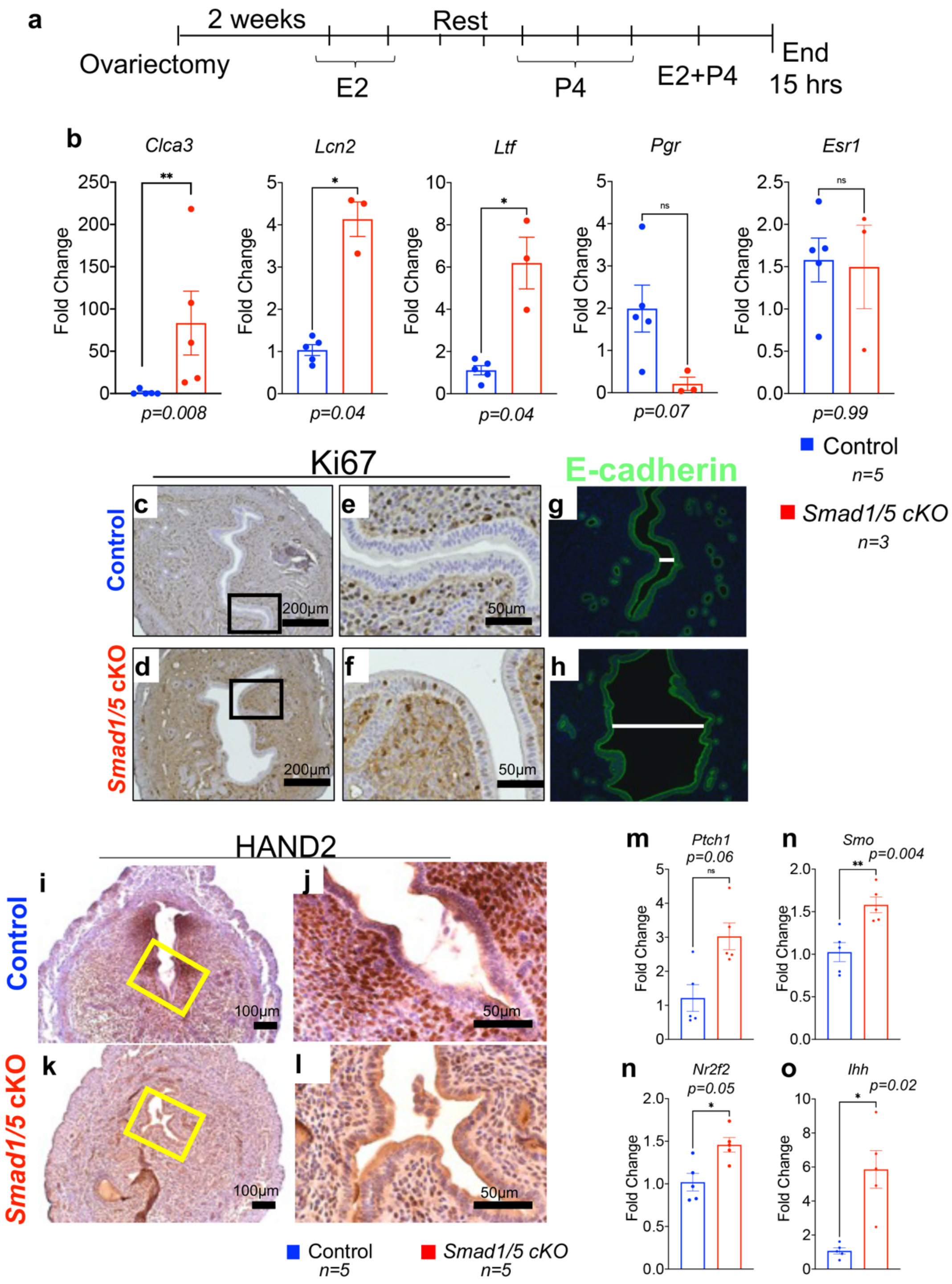

Supplementary Figure 2. Administration of exogenous progesterone does not attenuate endometrial estrogen-induced gene expression, proliferation, or luminal closure and implantation marker analysis at 4.5dpc. a) Experimental scheme to study the endometrial response to exogenous hormones in mice **b**) qPCR analysis in the endometrial epithelium of control (blue bars, n=5) and *Smad1/5* cKO (red bars, n=3) 15 hours after administration of E2+P4 (10ng E2+1mg P4). Histograms represent mean ± standard error of the mean (SEM), Mann-Whitney, two-tailed test, *p<0.033,

p<0.002, ***P<0.001. **c-h) Histological analysis of the uterus from controls (**c, e, g**) and *Smad1/5* cKO (**d, f, g**) mice 15 hours after administration of E2+P4. Ki67 staining in control (**c,e**) and *Smad1/5* cKO (**d,f**); E-Cadherin (green) immunostaining in control (**g**) and *Smad1/5* cKO (**h**) tissues. White bar indicates absence of luminal closure in *Smad1/5* cKO mice (**h**). **i-l**) IHC of HAND2 in the uterine tissues of control (**i-j**) and *Smad1/5* cKO (**k-l**) mice. **m-o**) qPCR analysis of progesterone-regulated genes (*Nr2f2, Ihh, Ptch1, Smo*) in uteri from control (blue bars, n=5) and *Smad1/5* cKO mice (red bars, n=5). Histograms represent mean ± standard error of the mean (SEM), paired, two-tailed student's *t*-test, *p<0.033, **p<0.002, ***P<0.001. Micrographs in (**c-h**) and (**i-l**) are representative images observed in at least three different subjects per pregnancy timepoint.

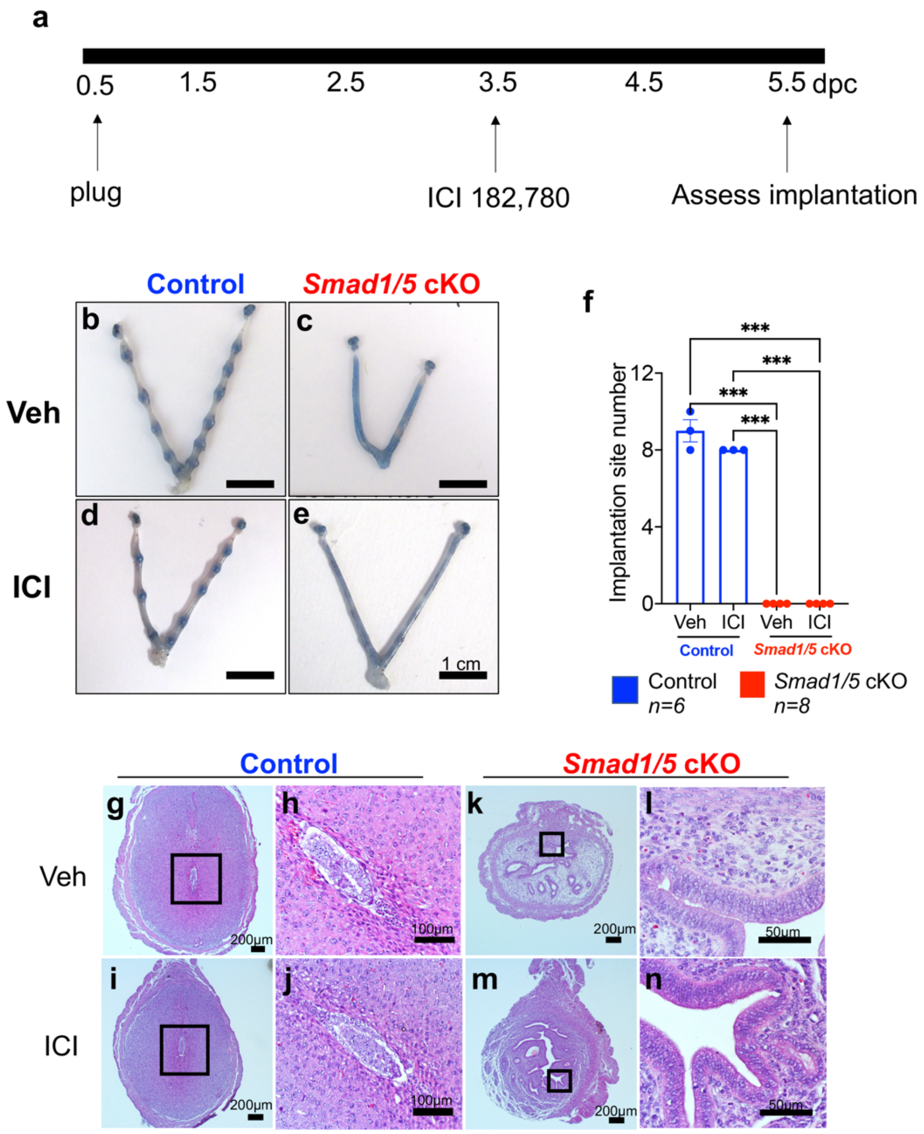

Supplementary Figure 3. Administration of an estrogen receptor antagonist, ICI 182,780, does not rescue implantation defect in *Smad1/5* cKO mice. a) Experimental scheme used to test the estrogen receptor antagonist, ICI 182, 780, in control and *Smad1/5* cKO mice. **b-e**) Gross images of the uterine tracts isolated from control (n=6, **b,d**) or *Smad1/5* cKO (n=8, **c-e**) mice at 5.5 dpc. Mice were treated with vehicle (Veh, **b,c**) or ICI 182, 780 (ICI, **d,e**). **f**) Quantification of the 5.5dpc implantation sites from the control (blue bars, n=6) or *Smad1/5* cKO (red bars, n=8) following vehicle or ICI 182, 780 administration. One-way *ANOVA*, p< 0.001. **g-n**) Histological analyses of the uterine tissues from control (**g-j**), or *Smad1/5* cKO (**k-l**) mice treated with vehicle (**g-h, k-l**) or ICI 182, 780 (**i-j, m-n**). Cross-sections were stained with Hematoxylin and Eosin (H&E). Images in (**g-n**) are representative of observations in at least three different subjects per condition. Histograms represent mean ± standard error of the mean (SEM), *p<0.033, **p<0.002, ***P<0.001.

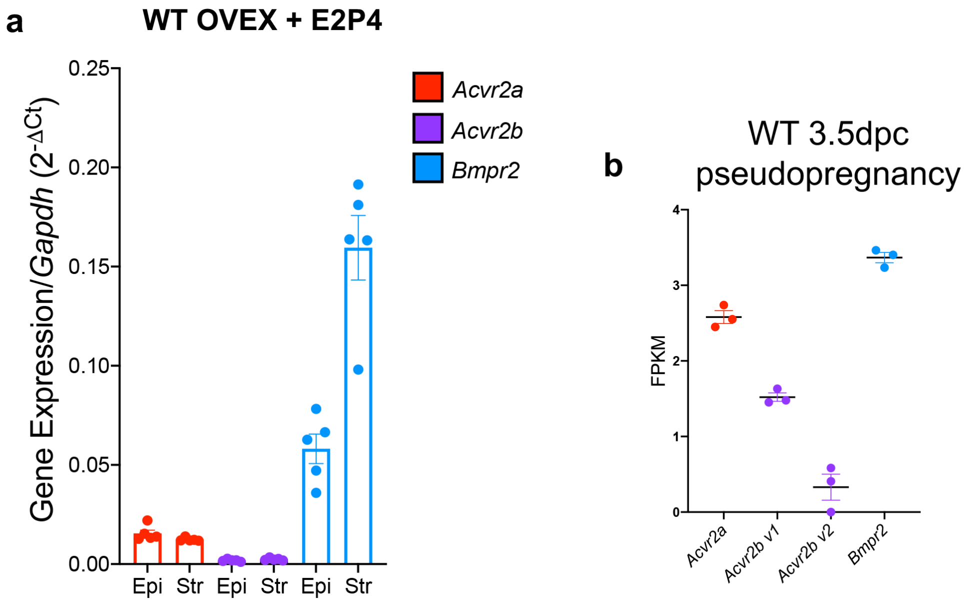

Supplementary Figure 4. BMP type 2 receptor expression in WT uterus. a) WT mice were ovariectomized and treated with hormones to mimic early pregnancy (as outlined in Supplementary Fig. 2A). Uterine tissues were collected 15 hours after the last E2+P4 injection (10ng E2+1mg P4) and the epithelium was enzymatically separated from the stroma. qPCR was used to detect the expression of *Acvr2a* (orange bars), *Acvr2b* (purple bars); *Bmpr2* (blue bars) in epithelium or stroma (n=5 mice). Histograms represent gene expression normalized to *Gapdh* plotted as mean ± SEM. **b**) RNAseq from WT mice at 3.5dpc of pseudopregnancy (n=3) showing the fragments per kilobase of exon per million reads (FKPM) for each *Acvr2a, Acvr2b* transcript variants 1 and 2 (v1, v2), and *Bmpr2.*

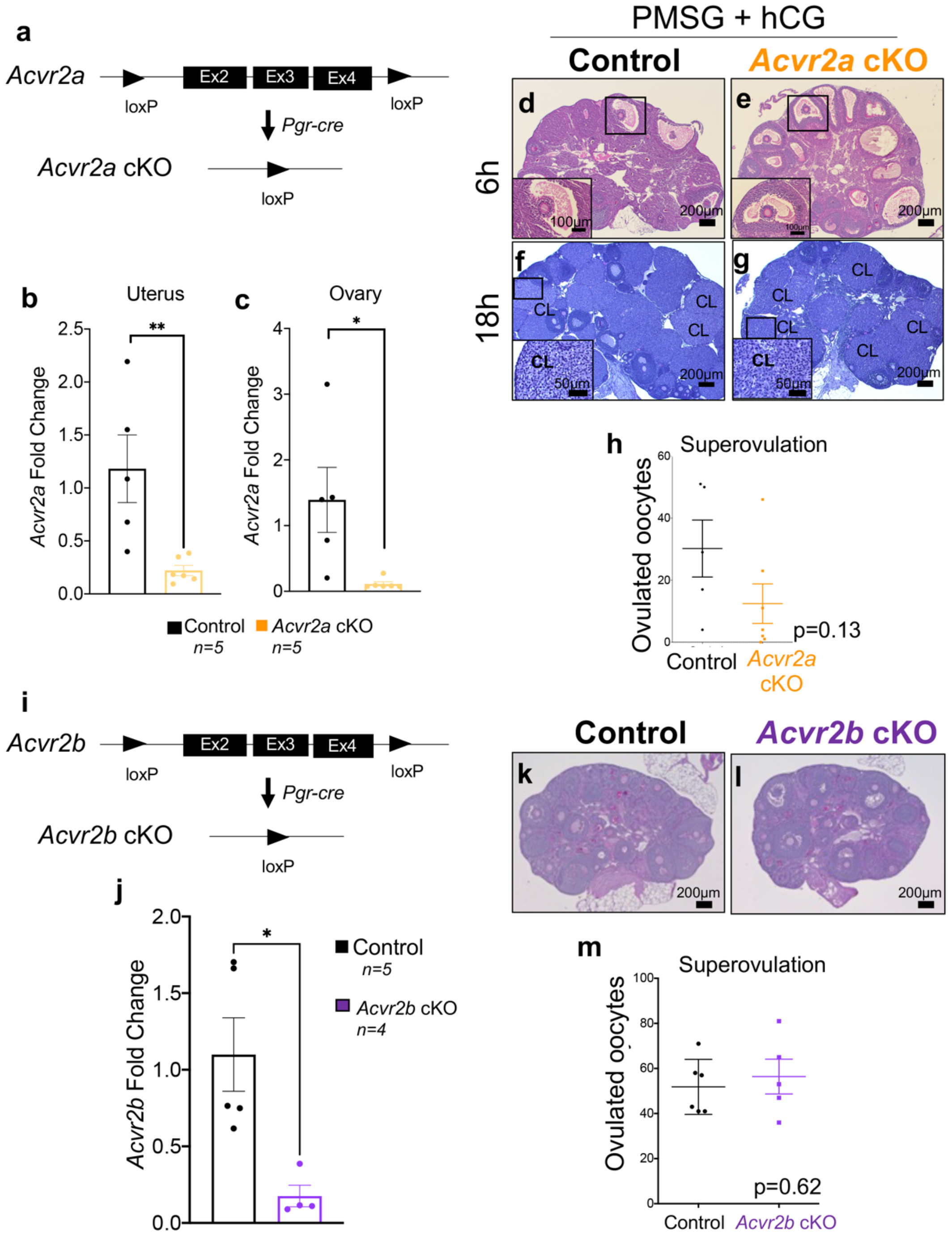

Supplementary Figure 5. Generation of *Acvr2a* cKO and *Acvr2b* cKO mice and analysis of ovulatory response. a) Conditional allele schematic for *Acvr2a,* shows that exons 2-4 are flanked by LoxP sites that are recombined by PR-cre in the *Acvr2a* cKO mice. **b-c**) qPCR analysis of the expression of *Acvr2a* exons 2-4 in the uterine *(p=0.01)* (**b**) and ovarian *(p=0.02)* (**c**) tissues of control (black bars, n=5) and *Acvr2a* cKO (orange bars, n=5) mice. Histograms represent mean ± standard error of the mean (SEM), paired, two-tailed student's *t*-test, *p<0.033, **p<0.002, ***P<0.001. **d-g**) PAS staining in the

ovarian cross-sections of control (**d,f**) and *Acvr2a* cKO (**e,g**) mice treated with PMSG + hCG. Ovaries were collected 6 hours after hCG administration to visualize pre-ovulatory follicles (**d-e**), or 18 hours after hCG administration to visualize corpora lutea (CL) in the ovaries (**f-g**). **h**) Ovulated oocytes were quantified in control (n=5) and *Acvr2a* cKO (n=5) mice following treatment with hCG+PMSG. Ovaries and oviducts were collected approximately 18 hours after hCG administration to quantify ovulated oocytes. **i**) Conditional allele schematic for *Acvr2b.* Exons 2-4 are flanked by LoxP sites that are recombined by PR-cre. **j**) qPCR analysis of exons 2-4 in the uterine tissues of control (black bars, n=5) and *Acvr2b* cKO (purple bars, n=4) mice, $p=0.01$. Mean ± standard error of the mean (SEM), unpaired, two-tailed student's *t*-test, *p<0.033, **p<0.002, ***P<0.001. **k-l**) PAS-stained cross-sections of ovaries from control (**k**) and *Acvr2b* cKO (**l**) mice. **m**) Ovulated oocytes collected from control and *Acvr2b* cKO mice treated with PMSG+hCG. Ovaries and oviducts were collected 18 hours after hCG administration to quantify ovulated oocytes. Mean ± SEM. Unpaired, two-tailed, student's *t*-test, *p<0.033, **p<0.002, ***P<0.001. Histology images represent experiments conducted in at least 3 subjects of each genotype.

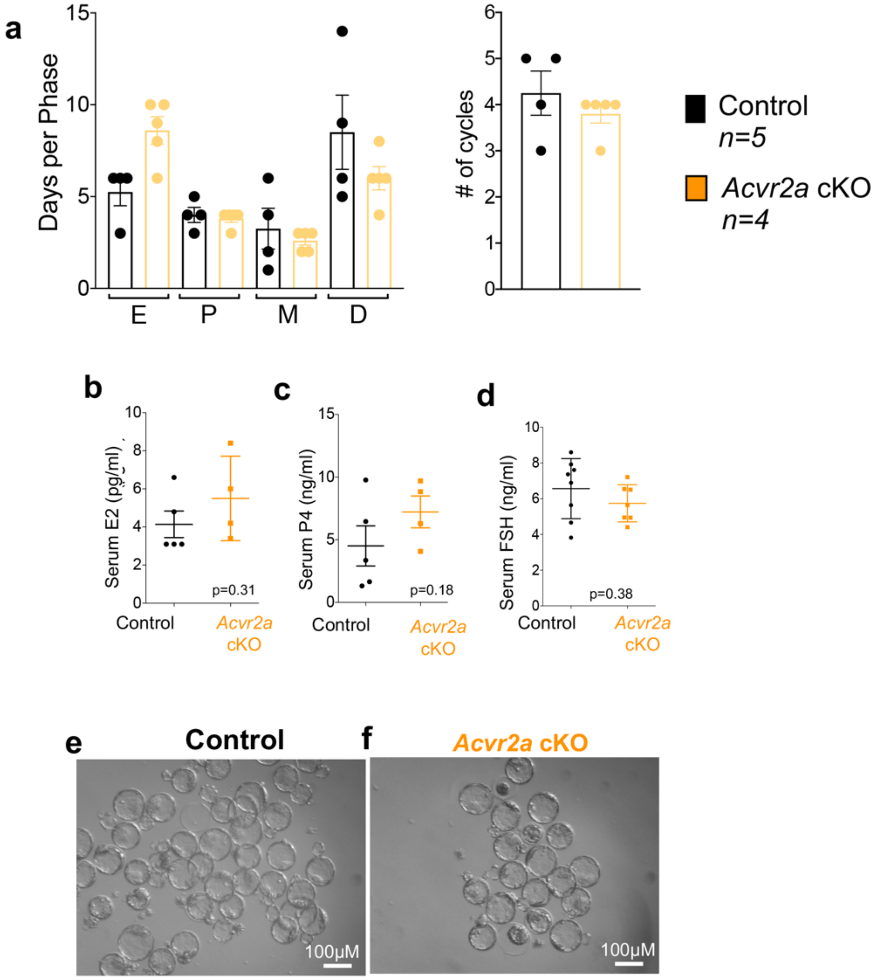

g

	**Control** ***n=6***	***Acvr2a* cKO** ***n=5***
**Oocytes**	214 ± 8.41	88 ± 3.38
**Zygotes**	199 ± 21.39	81 ± 4.66
**2-cell stage**	197 ± 18.48	78 ± 6.31
**Blastocysts**	166 ± 16.94	48 ± 6.95

Supplementary Figure 6. Analysis of fertilization and blastocyst development is normal in WT embryos from *Acvr2a* cKO females. a) Estrous phase analyses were performed for >21 days in control (black bars, n=5) and *Acvr2a* cKO (orange bars, n=4) mice. *E*= estrus, *P*= proestrus, *M*=metestrus, *D*=diestrus. **b-c**) Serum hormone analyses performed for estradiol (E2) (**b**) and progesterone (P4) (**c**) in a group of control (black dots, n=5) and *Acvr2a* cKO (orange dots, n=4) collected during estrus. **d**) Analysis of serum FSH was determined in control (black dots, n=8) and *Acvr2a* cKO (orange dots, n=7) mice collected during diestrus. Graphs represent mean ± SEM. **e-f**) Images of blastocysts from natural

fertilizations of control (**e**) or *Acvr2a* cKO (**f**) females mated to WT males. Images in (**e-f**) are representative images observed in 6 control and 5 *Acvr2a* cKO mice. **g**) Quantification of early embryonic development of fertilized oocytes collected at 0.5dpc from control (n=6) and *Acvr2a* cKO (n=5) females mated to WT males. Quantification of zygotes, 2-cell stage embryos, and blastocysts was performed daily. Estrous cycle histograms (**a**) represent mean ± SEM analyzed by one-way ANOVA, followed by a Tukey's multiple comparison post-hoc test. **b-c**) Analyzed by unpaired two-tailed, student's *t*-test. *p<0.033, **p<0.002, ***p<0.001.

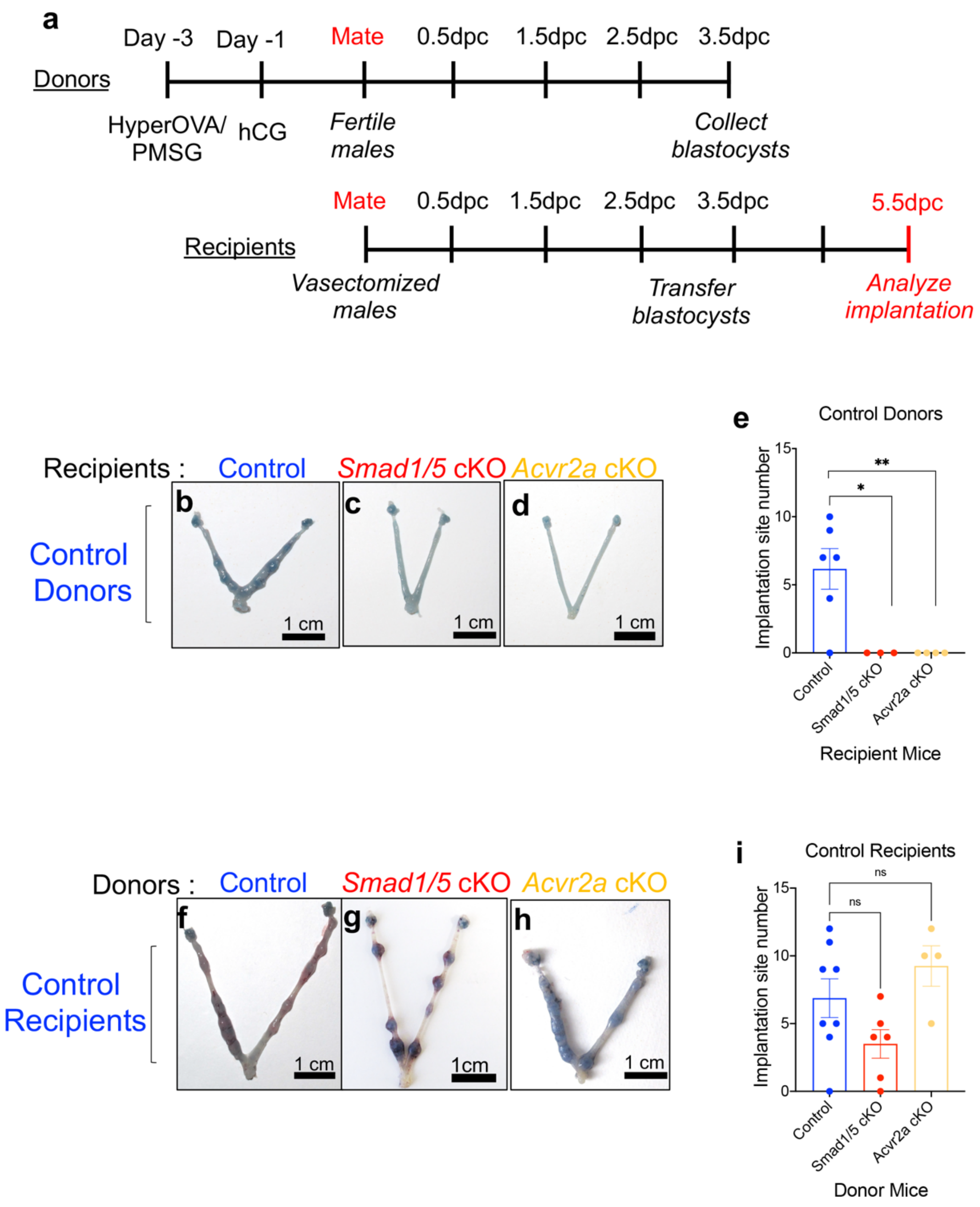

Supplementary Figure 7. Assessing implantation in control, *Smad1/5* cKO, and *Acvr2a* cKO mice by performing embryo transfers. a) Experimental schematic used to perform embryo transfer experiments in control, *Smad1/5* cKO and *Acvr2a* cKO mice. **b-d**) Representative images of gross uterine tissues isolated from control (**b**), *Smad1/5* cKO (**c**), and *Acvr2a* cKO (**d**) recipient mice that received embryos from control donors. **e**) Quantification of implanted embryos from control donors observed at 5.5dpc, p= 0.005. Mean ± SEM analyzed by one-way ANOVA, followed by a Dunnett's multiple comparison post-hoc test, *p<0.033, **p<0.002, ***p<0.001. **f-h**) Representative images of gross

uterine tissues isolated from control recipient mice (**f-h**) that received embryos from control (**f**), *Smad1/5* cKO (**g**), or *Acvr2a* cKO (**h**) donors. **i**) Quantification of the number of implantation sites observed in control mice from control, *Smad1/5* cKO or *Acvr2a* cKO donor mice, $p=0.05$. Mean ± SEM analyzed by one-way ANOVA, followed by a Dunnett's multiple comparison post-hoc test, $^{*}p<0.033$, $^{**}p<0.002$, $^{***}p<0.001$.

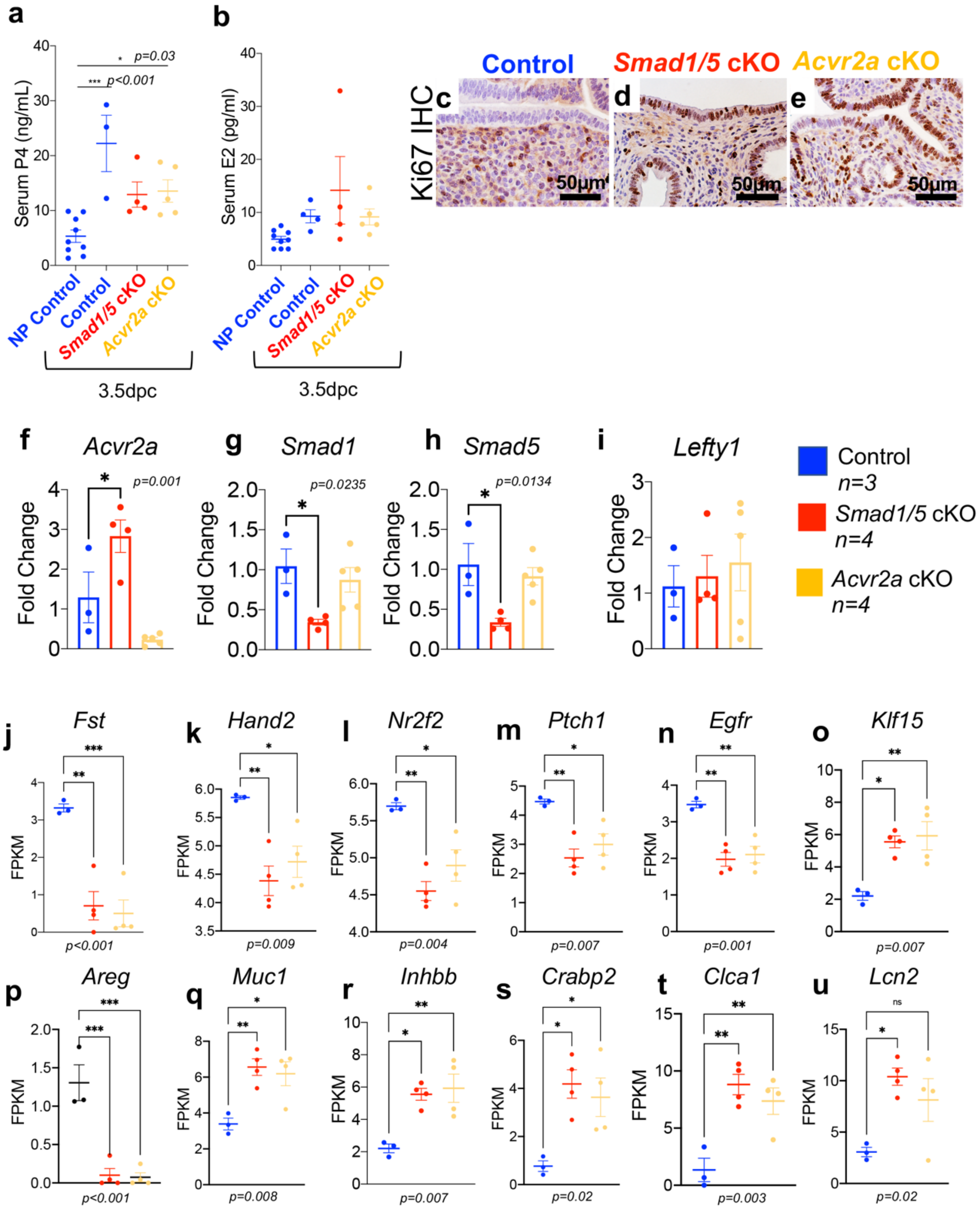

Supplementary Figure 8. Profiling the window of implantation in *Smad1/5* cKO and *Acvr2a* cKO mice by quantifying serum estrogen and progesterone and uterine gene expression patterns. a) Serum progesterone (P4) levels from non-pregnant controls (estrus phase), or 3.5dpc pseudopregnant control (blue dots), *Smad1/5* cKO (red dots), or *Acvr2a* cKO (yellow dots). **b**) Serum estradiol (E2) levels from non-pregnant controls (diestrus phase), or 3.5dpc control (blue dots), *Smad1/5* cKO (red dots), or *Acvr2a* cKO (yellow dots). **c-e**) Ki67 IHC in the uterine cross-sections isolated from 3.5dpc

pseudopregnant controls (**c**), *Smad1/5* cKO (**d**), or *Acvr2a* cKO (**e**) mice. IHC staining performed in at least 3 subjects per genotype. **f-i**) qPCR analysis of *Acvr2a, Smad1, Smad5* and *Lefty* in the 3.5dpc pseudopregnant tissues of control (n=3, blue bars), *Smad1/5* cKO (n=4, red bars), and *Acvr2a* cKO (n=4, yellow bars) mice. Histograms represent mean ± SEM analyzed by one-way ANOVA, followed by a Tukey's multiple comparison post-hoc test, *p<0.033, **p<0.002, ***p<0.001. **j-u**) Scatterplots showing the total number of reads (fragments per kilobase of transcript per million mapped reads, FPKM) in control (n=3, blue bars), *Smad1/5* cKO (n=4, red bars), and *Acvr2a* cKO (n=4, yellow bars) mice determined by RNAseq at 3.5dpc of pseudopregnancy. Plots represent mean ± SEM analyzed by one-way ANOVA, followed by a Tukey's multiple comparison post-hoc test, *p<0.033, **p<0.002, ***p<0.001.

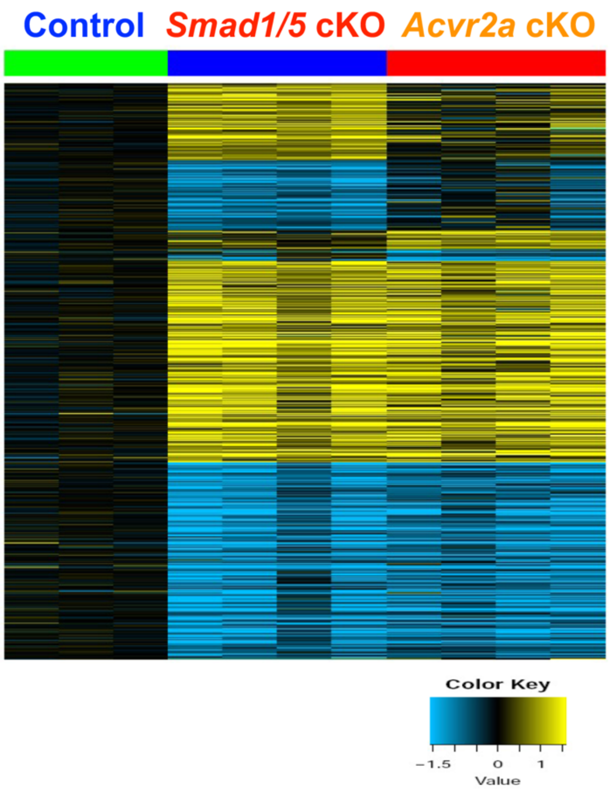

Supplementary Figure 9. RNA-Seq analysis of pseudopregnant 3.5dpc *Smad1/5* cKO and *Acvr2a* cKO mice reveals conserved gene expression pathways. Supervised hierarchical clustering of RNA-seq samples of control (n=3), *Smad1/5* cKO (n=4), and *Acvr2a* cKO (n=4) mice. Genes differentially expressed in either *Smad1/5* cKO or *Acvr2a* cKO groups (p<0.01 by t-test and fold change>1.4) are represented, 2112 genes in all. Bright yellow or blue represents a 3-fold change from the corresponding control group.

Supplementary Table 1. Fertility trial in *Smad1* cKO, *Smad5* cKO, *Smad1/5* cKO and *Acvr2a* cKO and *Acvr2b* cKO mice.

6-month fertility trial of *Smad1* cKO, *Smad5* cKO and *Smad1/5* cKO mice.

	Total Pups	Total Litters	Pups/Female	Pups/Litter	Avg. days to first litter
**Control (n=8)**	242	40	30.25 ± 4.17	6.08 ± 0.93	20 ± 1.83
***Smad1* cKO (n=8)**	205	31	25.63 ± 6.77	6.77 ± 1.16	24 ± 0.82
***Smad5* cKO (n=8)**	61	20	7.63 ± 4.92	3.06 ± 0.35	31 ± 12.36
***Smad1/5* cKO (n=8)**	0	0	0	0	-

6-month fertility trial of *Acvr2b* cKO and *Acvr2a* cKO mice.

	Total Pups	Total Litters	Pups/Female	Pups/Litter	Avg. days to first litter
**Control (n=10)**	564	67	56.4 ± 12.74	8.35 ± 1.51	24.9 ± 3.14
***Acvr2a* cKO (n=8)**	0	0	0	0	-
***Acvr2b* cKO (n=10)**	266	52	26.6 ± 9.33	5.17 ± 0.99	30.7 ± 15.83

Supplementary Table 2. List of primer sequences for genotyping and qPCR.

**Gene Expression Primers, 5'-3'**		
**Gene Symbol**	**Forward (5'-3')**	**Reverse (5'-3')**
Bmp2	GGGACCCGCTGTCTTCTAGT	TCAACTCAAATTCGCTGAGGAC
Ltf	TGAGGCCCTTGGACTCTGT	ACCCACTTTTCTCATCTCGTTC
Lcn2	GCAGGTGGTACGTTGTGGG	CTCTTGTAGCTCATAGATGGTGC
Muc1	GGCATTCGGGCTCCTTTCTT	TGGAGTGGTAGTCGATGCTAAG
Vim	TGCACGATGAAGAGATCCAGG	CTCCTGGAGGTTCTTGGCAG
Krt18	GAGGGCTCAGATCTTTGCGA	CATGGATGTCGCTCTCCACA
Ptgs2	AGCCAGGCAGCAAATCCTT	CAGTCCGGGTACAGTCACAC
Ereg	TGCTTTGTCTAGGTTCCCACC	GGCGGTACAGTTATCCTCGG
Wnt4	GAGAAGTGTGGCTGTGACCGG	ATGTTGTCCGAGCATCCTGACC
Klf4	GTGCCCCGACTAACCGTTG	GTCGTTGAACTCCTCGGTCT
Klf15	GAGACCTTCTCGTCACCGAAA	GCTGGAGACATCGCTGTCAT
Mcm2	ATCCACCACCGCTTCAAGAAC	TACCACCAAACTCTCACGGTT
Mcm7	AGTATGGGACCCAGTTGGTTC	GCATTCTCGCAAATTGAGTCG
Ptch1	AAAGAACTGCGGCAAGTTTTTG	CTTCTCCTATCTTCTGACGGGT
Smo	GAGCGTAGCTTCCGGGACTA	CTGGGCCGATTCTTGATCTCA
Nr2f2	TCAACTGCCACTCGTACCTG	CCATGATGTTGTTAGGCTGCAT
Ihh	CTCTTGCCTACAAGCAGTTCA	CCGTGTTCTCCTCGTCCTT
Ccnd1	CATCAAGTGTGACCCGGACTG	CCTCCTCCTCAGTGGCCTTG
Clca3	CTGTCTTCCTCTTGATCCTCCA	CGTGGTCTATGGCGATGACG
Sfrp1	CAACGTGGGCTACAAGAAGAT	GGCCAGTAGAAGCCGAAGAAC
Sfrp2	CGTGGGCTCTTCCTCTTCG	ATGTTCTGGTACTCGATGCCG
Sfrp3	ATTTGGTGTTCTGTACCCTG	CGTTTCCTCATAAAATGCTTC
Sfrp4	AGAAGGTCCATACAGTGGGAAG	GTTACTGCGACTGGTGCGA
Sfrp5	CACTGCCACAAGTTCCCCC	TCTGTTCCATGAGGCCATCAG
Smad1 Ex2	CCTGGACAGCCGAGTAACTG	ACTCACAGCATTCCAGAGGC
Smad5 Ex2	TGTTGGGCTGGAAACAAGGT	GTGACACACTTGCTTGGCTG
Acvr2a Ex2-4	CTTGGCAGATCAGAAACTCAGG	GAGTAGGAACAAGTACAGGAGG
Acvr2b Ex2-4	GTGCATCTACTACAACGCCA	GGAAGATCTTCACAGCCACAAAG
Pgr	CCCACAGGAGTTTGTCAAGCTC	TAACTTCAGACATCATTTCCGG
Esr1	GCTCCTAACTTGCTCCTGGAC	CAGCAACATGTCAAAGATCTCC
**Genotyping Primers, 5'-3'**		
PR cre WT	CCCAAAGAGACACCAGGAAG	
PR cre F	TATACCGATCTCCCTGGACG	
PR cre R	ATGTTTAGCTGGCCCAAATG	
ACVR2A_914	CCACTGATACCATTGTCACATGTTATCCTAATGCTAG	
ACVR2A_916	CCATTATGTAGAGTGCTGTCATTAGTTCAGTGCC	
ACVR2A_917	CTAAGAGACCCAGAAGGCCCAAGGTATTC	
ACVR2B_877	CAGGTGGGTTATTGGAGTAGGCTGGG	
ACVR2B_878	CACTCCACTGTGTCCAGGGGCTG	
ACVR2B_880	GATCTCTGGGGTAGCTGACAACAGCG	

Supplementary Table 3. List of antibodies and dilutions

Antibody	Company	Species	Cat #	IHC Dilution	Western Blot Dilution
E-Cadherin	Cell Signaling	Rabbit	3195	1/200	-
pSMAD1/5	Cell Signaling	Rabbit	13820S and 9516	1/200	1/1000
HAND2	R & D	Rabbit	AF3876-SP	1/200	
Progesterone Receptor	Cell Signaling	Rabbit	8757T	1/200	
KI67	BD Pharmingen	Mouse	550609	1/500	-
FOXA2	Abcam	Rabbit	ab108422	1/1000-1/5000	
ACVR2A	R&D	Goat	AF340-SP	1/1000	
Donkey anti-Rabbit-Alexa Fluor 488	Invitrogen	Donkey anti-Rabbit-	A21206	1/250	
Donkey anti-Mouse IgG-Alexa-Fluor 594	Invitrogen	Donkey anti-Mouse	A21203	1/250	
Donkey anti-Rat-Alexa-Fluor-594	Invitrogen	Donkey anti-Rat	A21209	1/250	
Peroxidase AffiniPure Donkey Anti-Rabbit	Jackson Immunoresearch	Donkey Anti-Rabbit-	711-035-152		1/5000
Goat Anti-Rabbit IgG Biotinylated	Vector Labs	Goat Anti-Rabbit	BA-1000	1/200	
Goat Anti-Rat IgG Biotinylated,	Vector Labs	Goat Anti-Rat	BA-9400	1/200	
Vector Mouse on Mouse Immunodetection Kit	Vector Labs		FMK-2201	Manufacturer's Recommendation	
