# Supplementary figures and images for: Endometrial receptivity and implantation require uterine BMP signaling through an ACVR2A-SMAD1/SMAD5 axis
Source: Nat Commun. 2021 Jun 7;12:3386. doi: 10.1038/s41467-021-23571-5 (PMC8184938; doi:10.1038/s41467-021-23571-5)

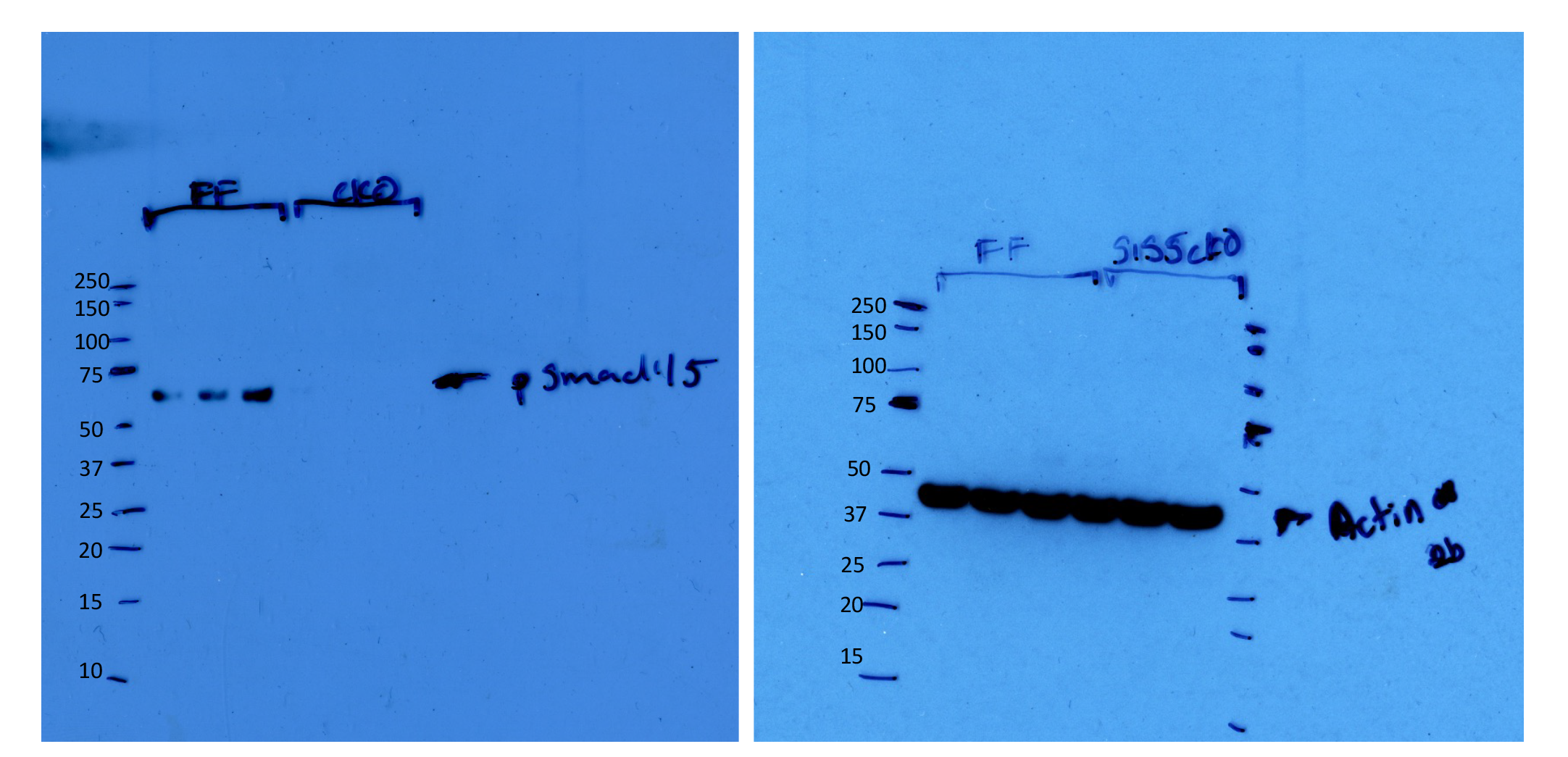

Supplement: Supplementary file 6 — Supplementary Data 5 [file 41467_2021_23571_MOESM6_ESM.png]
