# Supplementary material for: Endometrial receptivity and implantation require uterine BMP signaling through an ACVR2A-SMAD1/SMAD5 axis
Source: Nat Commun. 2021 Jun 7;12:3386. doi: 10.1038/s41467-021-23571-5 (PMC8184938; doi:10.1038/s41467-021-23571-5)
Supplement: Supplementary file 7 — Reporting Summary [file 41467_2021_23571_MOESM7_ESM.pdf]

nature research

Corresponding author(s): Diana Monsivais and Martin M. Matzuk

Last updated by author(s): Apr 25, 2021

Reporting Summary

Nature Research wishes to improve the reproducibility of the work that we publish. This form provides structure for consistency and transparency in reporting. For further information on Nature Research policies, see our Editorial Policies and the Editorial Policy Checklist.

Statistics

For all statistical analyses, confirm that the following items are present in the figure legend, table legend, main text, or Methods section.

n/a	Confirmed	
☐	☒	The exact sample size (n) for each experimental group/condition, given as a discrete number and unit of measurement
☐	☒	A statement on whether measurements were taken from distinct samples or whether the same sample was measured repeatedly
☐	☒	The statistical test(s) used AND whether they are one- or two-sided<br>*Only common tests should be described solely by name; describe more complex techniques in the Methods section.*
☒	☐	A description of all covariates tested
☒	☐	A description of any assumptions or corrections, such as tests of normality and adjustment for multiple comparisons
☐	☒	A full description of the statistical parameters including central tendency (e.g. means) or other basic estimates (e.g. regression coefficient) AND variation (e.g. standard deviation) or associated estimates of uncertainty (e.g. confidence intervals)
☐	☒	For null hypothesis testing, the test statistic (e.g. F, t, r) with confidence intervals, effect sizes, degrees of freedom and P value noted<br>*Give P values as exact values whenever suitable.*
☒	☐	For Bayesian analysis, information on the choice of priors and Markov chain Monte Carlo settings
☒	☐	For hierarchical and complex designs, identification of the appropriate level for tests and full reporting of outcomes
☒	☐	Estimates of effect sizes (e.g. Cohen's d, Pearson's r), indicating how they were calculated

Our web collection on statistics for biologists contains articles on many of the points above.

Software and code

Policy information about availability of computer code

Data collection	HISAT2 and cufflinks (PMID 31375807) programs were used to align sequencing reads from RNAseq studies. Images from OPT and multiphoton micrsocope were analyzed with NRECON and Imaris software.
Data analysis	Sequences from RNA sequencing studies were aligned and transcript abundance was performed using HISAT2 2.1.0 and cufflinks 2.2.1.2: PMID 31375807<br>Gene ontology enrichment analysis was performed on all the up- and down-regulated genes using the Sigterms program v1.0: PMID 18812437<br>Further analysis and determination of the redundancy of duplicated terms was performed with the REVIGO program v1.0 using an allowed similarity score of small (0.5): PMID 21789182<br>For reconstructing OPT and multiphoton microscopy images, Imaris 9.2.1 and NRecon 1.7.1.0 were used.

For manuscripts utilizing custom algorithms or software that are central to the research but not yet described in published literature, software must be made available to editors and reviewers. We strongly encourage code deposition in a community repository (e.g. GitHub). See the Nature Research guidelines for submitting code & software for further information.

Data

Policy information about availability of data

All manuscripts must include a data availability statement. This statement should provide the following information, where applicable:

- Accession codes, unique identifiers, or web links for publicly available datasets
- A list of figures that have associated raw data
- A description of any restrictions on data availability

The following data availability statement is included in the manuscript:

"The datasets generated in this study are available in the Gene Expression Omnibus Accession Code GSE152675 or by contacting the corresponding authors."

Field-specific reporting

Please select the one below that is the best fit for your research. If you are not sure, read the appropriate sections before making your selection.

- [x] Life sciences
- [ ] Behavioural & social sciences
- [ ] Ecological, evolutionary & environmental sciences

For a reference copy of the document with all sections, see nature.com/documents/nr-reporting-summary-flat.pdf

Life sciences study design

All studies must disclose on these points even when the disclosure is negative.

Sample size

For our mouse studies, the sample size was determined based on a power analysis calculation that took into account various paraments, such as effect size, standard deviation, type 1 error and direction of effect. Because of the genetic nature of our animal studies, and the limitations based on sex and age, we also factored an expected attrition of animals in our breeding schemes.
For the experiments other than those involving mice, a minimum sample size of three was utilized to ensure that findings were consistent across a variety of technical and biological conditions. If findings across the samples were found to be consistent, and that the experimental assumptions were held constant, this was determined to be sufficient sample size. This approach was necessary in the experiments requiring specimens from human donors, where the availability of samples was extremely limited.

Data exclusions

For pregnancy studies, only data from all mice deemed to be at the correct stage of pregancy were utilized (based on serum levels of progesterone), or based on the number of blastocysts recovered after flushing of the uterus. Those samples from mice that were deemed not to be at the correct stage of pregnancy were excluded from analysis.

Replication

To verify the reproducibility of our studies, experiments were repeated in samples from various subjects and performed on different dates, often by different investigators.
Obtaining mice of the same age, sex, and desired genotypes that are at the specific time of pregnancy, required various rounds of mating and timed-mating. Therefore, to obtain enough mice of the correct genotype and stage of pregnancy, the experiments were carried out over the course of at least 6 months, with various rounds of mouse breeding, genotyping, timed mating, dissection and verification of pregnancy. Once the required cohort of mice was obtained, the desired endpoints were assessed and replicated at minimum 3 times. These attempted replicates were successful, given that the techniques assessed are well-established in our laboratory and routinely performed by all lab-members. If unsuccessful attempts were obtained, this was attributed to the absence of a pregnancy, or an incorrectly staged animal.

Randomization

Samples and organisms were separated into specific groups based on 1) genotype, 2) pregnancy status, 3) or estrous cycles. Because studies were investigating early pregnancy, only female mice were utilized.

Blinding

Because the analysis was being performed on grouped mice according to genotype, and the differences in gene expression and histology were based on these genetic differences and pregnancy states, blinding was not performed

Reporting for specific materials, systems and methods

We require information from authors about some types of materials, experimental systems and methods used in many studies. Here, indicate whether each material, system or method listed is relevant to your study. If you are not sure if a list item applies to your research, read the appropriate section before selecting a response.

Materials & experimental systems

n/a	Involved in the study	
[ ]	[x]	Antibodies
[x]	[ ]	Eukaryotic cell lines
[x]	[ ]	Palaeontology and archaeology
[ ]	[x]	Animals and other organisms
[ ]	[x]	Human research participants
[x]	[ ]	Clinical data
[x]	[ ]	Dual use research of concern

Methods

n/a	Involved in the study	
[x]	[ ]	ChIP-seq
[x]	[ ]	Flow cytometry
[x]	[ ]	MRI-based neuroimaging

Antibodies

Antibodies used

E-Cadherin, Cell Signaling #3195; phosphoSMAD1/5, Cell Signaling #13280 and 9516; HAND2, R&D Systemsm # AF3876-SP; Progesterone receptor, Cell Signaling, #8757T; Ki67, BD Pharmingen, #550609; FOXA2, Abcam, #ab108422; ACVR2A, R&D Systems, #AF340-SP. Secondary antibodies for Immunoflourescence: Donkey anti-Rabbit IgG (H+L) Highly Cross-Adsorbed Secondary Antibody, Alexa Fluor 488, Invitrogen #A21206; Donkey anti-Mouse IgG (H+L) Highly Cross-Adsorbed Secondary Antibody, Alexa Fluor 594, Invitrogen #A21203; Donkey anti-Rat IgG (H+L) Highly Cross-Adsorbed Secondary Antibody, Alexa Fluor 594, Invitrogen #A21209.

	Secondary antibodies for western blot: Peroxidase AffiniPure Donkey Anti-Rabbit IgG (H+L), Jackson ImmunoResearch 711-035-152. Biotinylated secondary antibodies for immunohistochemistry: Goat Anti-Rabbit IgG Biotinylated , Vector Biolabs, BA-1000; Goat Anti-Rat IgG Biotinylated, Vector Biolabs, BA-9400, Vector Mouse on Mouse Immunodetection Kit, Vector Biolabs, FMK-2201.
Validation	1) E-Cadherin, Cell Signaling #3195: specificity validated by manufacturer as indicated on their website by IHC, western blot, immunoflourescence and flow cytometry. This antibody is highly specific and reliable and has 1,328 citations listed on its website.<br>2) phosphoSMAD1/5, Cell Signaling #13820 and 9516: specificity validated by manufacturer as listed on their website (IP, western blot, immunoflourescence and flow cytometry). These antibodies were also validated by by our group on tissues from knockout mice. The antibodies are highly reliable and have been cited highly in the literature: 222 citations (9516) and 145 (13820).<br>3) HAND2, R&D Systemsm # AF3876-SP: HAND2 specificity has been validated by the manufacturer in direct ELISAs and in Western blots. We observed the specificity of this antibody in the decidualizing endometrial stroma during implantaiton, as previously reported (PMID 21330545).<br>4) Progesterone receptor, Cell Signaling, #8757T: Validated by the manufacturer on western blots, IHC, IF, ChIPseq, and flow cytometry. Antibody is highly specific and has 33 listed citations.<br>5) Ki67, BD Pharmingen, #550609: Validated by the manufacturer using western blot analysis of proliferating cells, flow cytometry and IHC of frozen and paraffin embedded tissues. In our studies, this antibody is highly specific. The website lists 14 references for this antibody.<br>6) FOXA2, Abcam, #ab108422: antibody was validated by the manufacturer using IHC, IF, and western blot. We also observe highly specific staining directed to the endometrial glands of the uterus. According to the manufacturer, this antibody has 45 references.<br>7) ACVR2A, R&D Systems, #AF340-SP: antibody is validated by the manufacturer using IHC, and is validated in direct ELISAs and western blots. The antibody has also 6 citations and was very specific to the decidualizing and epithelial cells of the pregnant uterus in our experiments.<br>8) Secondary antibodies for immunofluorescence (Donkey anti-Rabbit IgG (H+L) Highly Cross-Adsorbed Secondary Antibody, Alexa Fluor 488, Invitrogen #A21206; Donkey anti-Mouse IgG (H+L) Highly Cross-Adsorbed Secondary Antibody, Alexa Fluor 594, Invitrogen #A21203; Donkey anti-Rat IgG (H+L) Highly Cross-Adsorbed Secondary Antibody, Alexa Fluor 594, Invitrogen #A21209): antibody specificity was validated by using a secondary-only control in the absence of a primary antibody. This allows visualization of non-specific binding.<br>9) Secondary antibody used for western blot (Jackson ImmunoResearch 711-035-152) is highly cited (622 citations listed on Citeab) and is highly specific in the mouse uterine tissue lysates used.<br>10) Secondary antibodies used for immunohistochemistry (Goat Anti-Rabbit IgG Biotinylated , Vector Biolabs, BA-1000; Goat Anti-Rat IgG Biotinylated, Vector Biolabs, BA-9400, Vector Mouse on Mouse Immunodetection Kit, Vector Biolabs, FMK-2201): antibody specificity was assessed by incubating tissue sections with a secondary-only (no primary antibody) control. These antibodies are widely cited in the literature, for example, according to CiteAb, BA-1000 has >2,000 citations and BA-9400 has 194 citations. The Vector Labs website states that FMK-2201 has been cited 258 times.

Animals and other organisms

Policy information about studies involving animals; ARRIVE guidelines recommended for reporting animal research

Laboratory animals	Mice were maintained on a hybrid C57BL/6J and 129S5/SvEvBrd genetic background. For all experiments, mice 6-8-weeks old were used unless specifically noted. Experimental mice were all female, given that the studies focused on pregnancy, uterine or ovarian function. Male mice were only utilized for breeding purposes or for inducing pregnancy state in the experimental female mice.
Wild animals	No wild animals were used.
Field-collected samples	This study did not involve studies from the field.
Ethics oversight	Animal handling and experimental studies were performed following the NIH Guide for the Care and Use of Laboratory Animals and were approved by the Institutional Animal Care and Use Committee of Baylor College of Medicine.

Note that full information on the approval of the study protocol must also be provided in the manuscript.

Human research participants

Policy information about studies involving human research participants

Population characteristics	Specimens were obtained from a human tissue bank at Baylor College of Medicine after receiving approval from the Institutional Review Board. Uterine specimens were from women of reproductive age (18-45) and were histologically staged to the specific phase of the menstrual cycle by a pathologist.
Recruitment	This study utilized specimens from a tissue bank, approved by and registered under the Institutional Review Board at Baylor College of Medicine, protocol number: H-46538 and H-211138.
Ethics oversight	Human endometrial tissues were obtained using protocols approved by the Institutional Review Board at Baylor College of Medicine and University of North Carolina School of Medicine (H-46538, H-211138).

Note that full information on the approval of the study protocol must also be provided in the manuscript.
