# Supplementary material for: Endometrial receptivity and implantation require uterine BMP signaling through an ACVR2A-SMAD1/SMAD5 axis
Source: Nat Commun. 2021 Jun 7;12:3386. doi: 10.1038/s41467-021-23571-5 (PMC8184938; doi:10.1038/s41467-021-23571-5)
Supplement: Supplementary file 8 — Description of Additional Supplementary Files [file 41467_2021_23571_MOESM8_ESM.pdf]

Title: Supplementary Data 1

Description: All differentially expressed transcripts identified by RNAseq in the uterus of Smad1/5 cKO and Acvr2a cKO at day 3.5 of pseudopregnancy. >1.4-fold, <0.6-fold, p<0.01

Title: Supplementary Data 2

Description: Shared differentially expressed transcripts identified by RNAseq in the uterus of Smad1/5 cKO and Acvr2a cKO at day 3.5 of pseudopregnancy. >1.4-fold, <0.6-fold, p<0.01

Title: Supplementary Data 3

Description: Selected differentially expressed genes in the uterus of Smad1/5 cKO and Acvr2a cKO mice at pseudopregnancy day 3.5. Genes are categorized by functional pathway.

Title: Supplementary Data 4

Description: Gene ontology analysis of differentially expressed genes in Smad1/5 cKO and Acvr2a cKO uterus by RNAseq.

Title: Supplementary Data 5

Description: Uncropped blots to accompany Supplementary Figure 1j.
